# Natural Killer Activity and Its Changes among Participants in a Smoking Cessation Intervention Program — a Prospective Pilot Study of 6 Months’ Duration

**DOI:** 10.2188/jea.11.238

**Published:** 2007-11-30

**Authors:** Akiko Ioka, Masakazu Nakamura, Noriko Shirokawa, Tomoko Kinoshita, Shizuko Masui, Kazue Imai, Kei Nakachi, Akira Oshima

**Affiliations:** 1 The Osaka Cancer Prevention and Detection Center.; 2 Saitama Cancer Center Research Institute.; 3 Department of Epidemiology, Radiation Effects Research Institute.; 4 Department of Cancer Control and Statistics, Osaka Medical Center for Cancer and Cardiovascular Diseases.

**Keywords:** natural killer activity, smoking cessation, age, urine cotinine

## Abstract

We examined the effect of smoking cessation on natural killer (NK) activity of peripheral blood lymphocytes in terms of a prospective study of 27 Japanese subjects who participated in a smoking cessation intervention program. This program was delivered by means of group-counseling offering 7 sessions of about 2 hours over 6 months to help smokers to discontinue the habit. Thirteen subjects ceased smoking (quitters), while 14 continued to smoke (cigarette smokers). NK activity before the intervention was correlated positively with age (correlation coefficient=0.46, P<0.05). NK activity remained almost constant among quitters, comparing the activity before and after the intervention, while it decreased among cigarette smokers although it was not statistically significant. In the subgroup analysis, NK activity increased among those aged less than 65 years, or urine cotinine levels over 800 ng/ml before the intervention, especially among quitters, but there were no statistical significances. Multiple regression analysis showed changes in NK activity were correlated significantly only with age (standard regression coefficient=-0.44, P<0.05). These findings suggest that smoking cessation intervention programs might have been more effective for younger than elder subjects in consideration of NK activity.

## INTRODUCTION

Cigarette smoking increases the risk for a variety of cancer^1^^)^, and smoking patients with cancer of the mouth, oro^-^, or hypopharynx were reported to be at a higher risk for second primary cancers than the non-smoking patients^2^^)^, while smoking cessation has effects such as decreased risk of them. Thus, altered cigarette status may induce changes in susceptibility to cancer development.

Cigarette smoking has been suggested to modulate the immune system, being in part responsible for an enhanced risk of cancer among smokers. Natural killer (NK) cells, a subpopulation of lymphocytes belonging to a non-specific arm, play an important role in the immune surveillance against tumor development, which are well documented in animal and human studies, such as a higher incidence of cancer among beige mice which had a selective deficiency in NK cell function^3^^)^, patients with Chediak-Higashi^4^^, ^^5^^)^ or X-linked lymphoproliferative syndrome^6^^)^ who had abnormalities of NK cell function. Very recently, individuals with low NK activity of peripheral blood lymphocytes in a general population were reported by two of the authors (K. N and K. I) to have an enhanced risk of cancer, in terms of a prospective cohort study^7^^)^. They used peripheral blood lymphocytes as effector cells since other lymphocytes such as NKT cells also have an NK activity. Cigarette smoking, as well as demographic characteristics, have also been found to influence NK activity; NK activity is reduced among cigarette smokers^8^^-^^11^^)^. On the other hand, smoking cessation was reported to increase NK activity^12^^, ^^13^^)^. But it is largely unknown the association between postcessation changes in NK activity and demographic characteristics or various smoking parameters at baseline. We therefore tried to clarify the effects of smoking cessation on NK activity through a prospective follow-up study.

## MATERIALS AND METHODS

### Subjects

Eligible study subjects were 45 community-based participants who applied for smoking cessation intervention programs carried out in six public health centers, Osaka prefecture. The intervention program was delivered by means of group-counseling offering 7 sessions of about 2 hours over 6 months to help smokers to discontinue the habit. After informed consent was obtained from all subjects, peripheral blood and urine samples were collected between one and two o’clock in the afternoon before starting the intervention program, and also at six months after the first collection, disregarding the success or failure of quitting smoking among subjects. All subjects completed a self-administered questionnaire on cigarette smoking, history of diseases, and use of medicaments. We defined quitting smoking as urine cotinine levels below 50 ng/ml at the second collection, being assessed with liquid chromatography, since cotinine is a main proximate metabolite of nicotine; in general, urine cotinine levels are well correlated with plasma cotinine concentrations^14^^)^. Cigarette smokers were defined as subjects who continued to smoke, and quitters are those who had ceased to smoke cigarette in our smoking cessation intervention for six months. Of 45 subjects, 18 subjects were excluded from the analysis, since they experienced serious events on health during this intervention, e. g., onset of infectious diseases, introduction of medications, and recovery from diseases. Thus, the data sets were obtained from 14 of 21 cigarette smokers (66.7%), and 13 of 24 quitters (54.2%).

### Assay of NK activity

The cytotoxic activity of peripheral blood lymphocytes was measured by ^51^Cr-release assay. The effector cells were obtained from 5 ml of heparinized peripheral blood samples by density gradient centrifugation on Conray-Ficoll mixtures. Target cells were K-562, a human myeloid leukemia cell line, and labeled with ^51^Cr. The effector / target cell ratio (E/T) was 20 / 1; both cells were co-incubated in 5% CO_2_ for 3.5 hr. Radioactivity was then counted with a gamma counter. NK activity as percentage specific lysis was determined according to the standard formula^7^^)^.

### Statistical methods

Spearman’s correlation coefficients were calculated for NK activity, age and urine cotinine levels before the intervention. Wilcoxon Rank Sum Test was used for comparing differences in NK activity and its changes (“after” minus “before” smoking cessation intervention) between two groups. Wilcoxon Signed Rank Test was carried out in NK activity between before and after the intervention among cigarette smokers and quitters. Multiple regression analysis was carried out to control possible confounding factors to examine associations between its changes and explanatory variables (i.e., age, smoking status, and urine cotinine levels before the intervention). Differences were considered significant if the P value was less than 0.05. Statistical package software, SPSS was used for statistical analysis.

## RESULTS

**Figure 1.**
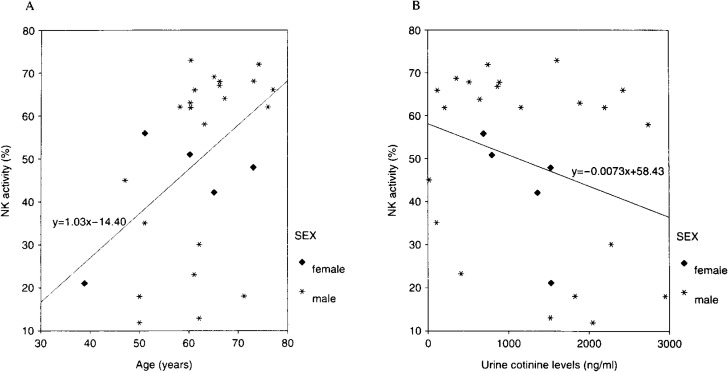
The relationship between NK activity and age (A) or urine cotinine levels (B) before the intervention (spearman’s correlation coefficient 0.46, P<0.05 or -0.26, P=0.19 for A or B, respectively).

The details of cigarette smokers and quitters were summarized in Table 1. Thirteen subjects ceased smoking (quitters), while 14 continued to smoke (cigarette smokers). Although there were not significant differences, the age distribution among quitters was younger than that among cigarette smokers, and urine cotinine levels before the intervention among quitters were lower than those among cigarette smokers. There were no remarkable differences in any characteristics between subjects included for analyses and those excluded (data not shown). Before smoking cessation intervention, NK activity at baseline was correlated positively with age (correlation coefficient=0.46, P<0.05) but not correlated significantly with urine cotinine levels (Figure 1). There was not remarkable sex difference in NK activity. Comparing NK activity before and after intervention, there were no statistically significant differences between cigarette smokers and quitters.

**Table 1.** Characteristics of participants in cigarette smokers and quitters at baseline.

	Cigarette Smokers (No. of cases=14; male=10, female=4)			Quitters (No. of cases=13; male=12, female=1)			P-value for difference ^a)^
	
	means	SE	median	means	SE	median	
Age (years)	63.0	2.3	63.5	60.5	2.9	61.0	n.s.
Cigarettes/day	20.4	2.6	20.0	18.2	3.0	17.0	n.s.
Smoking index	757.9	102.7	775.0	738.8	150.1	510.0	n.s.
Urine cotinine at baseline (ng/ml)	1289.9	176.0	1459.5	1203.6	290.0	893.0	n.s.
NK activity at baseline (%)	50.5	5.4	56.5	48.1	5.9	58.0	n.s.

^a)^ Wilcoxon Rank Sum Test is used for comparing differences in characteristics between cigarette smokers and quitters.

Table 2 shows the changes between NK activity before and after the intervention among cigarette smokers and quitters, categorizing them into two different grades in age, the number of cigarettes consumed per day, smoking index and urine cotinine levels before the intervention. Smoking index is defined as the number of cigarettes consumed per day × years. NK activity decreased among cigarette smokers, and remained almost constant among quitters. NK activity of quitters increased from the baseline levels, specifically among those with less than 65 years old, less than 20 cigarettes consumed per day, less than 500 smoking index, or 800 ng/ml urine cotinine and over. In contrast, NK activity of cigarette smokers decreased from the baseline levels during the same period with all categories except that of less than 500 smoking index. Statistically significant decreases in NK activity were observed among cigarette smokers with 65 years old and over, with 500 smoking index and over, and quitters with 20 cigarettes consumed per day and over. When we compared the changes of NK activity between cigarette smokers and quitters, there were no statistical significances in any category.

**Table 2.** Changes between NK activity before (baseline) and after the intervention among cigarette smokers or quitters.

		No. of cases	Cigarette Smokers	Quitters	P-value for difference ^b)^
Age	<65	16	-2.0± 5.9 ^a)^	4.1± 8.8	n.s.
	≧65	11	-18.2± 5.8 ^c)^	-8.3± 5.9	n.s.
Cigarettes/day	<20	12	-4.2± 5.3	10.7± 9.4	n.s.
	≧20	15	-13.4± 6.4	-11.8± 6.0 ^c)^	n.s.
Smoking index	<500	9	0.3± 8.0	4.5± 8.3	n.s.
	≧500	18	-13.0± 5.2^c)^	-3.2±10.0	n.s.
Urine cotinine (ng/ml) ^d)^	<800	10	-15.0±12.1	-3.3± 2.0	n.s.
	≧800	17	-8.2± 4.6	3.4±12.1	n.s.
Changes in NK activity (%)	All subjects	27	-10.1± 4.6	0.3± 6.4	n.s.

^a)^ Data are presented as means and standard errors.

^b)^ Wilcoxon Rank Sum Test is used for comparing differences in characteristics between cigarette smokers and quitters.

^c)^ After the smoking cessation intervention, there was statistically significant decrease by Wilcoxon Signed Rank Test analysis. P<0.05 (P values assessed in two tailed test).

^d)^ Measured before the intervention.

In multiple regression analysis, standard regression coefficients were calculated to assess the degree to which its changes were associated with age, smoking status, and urine cotinine levels before the intervention (Table 3). The changes of NK activity before and after intervention were significantly correlated only with age (standard regression coefficient=-0.44, P<0.05). This correlation was mainly attributed that among quitters, since cigarette smokers showed much less correlation (standard regression coefficient=-0.36, P=0.21).

**Table 3.** Multiple regression coefficients between changes of NK activity and explanatory variables (i.e., ages, smoking status, and urine cotinine before the intervention).

Explanatory variable	Regression coefficient	Standard regression coefficient	P-value for difference
Age	-0.97	-0.44	p<0.05
Urine cotinine	5.08 × 10^-3^	0.21	n.s.
Smoking status ^a)^	-8.42	-0.21	n.s.

^a)^ Smoking status is dummy variable: “0” and “1” are defined as quitting smoking and continuing to smoke.

## DISCUSSION

Hersey et al reported that there was a significant increase in NK cell activity among 35 subjects between 17 and 55 years of age who had ceased to smoke cigarette for three months compared with cigarette smokers^12^^)^, and Meliska et al also reported that abstinence increased NK cell cytotoxic activity among 28 subjects between 21 and 35 years of age who had ceased to smoke cigarette for 31 days^13^^)^. However we could not confirm their findings. Supplemental analysis of our study indicated that changes in NK activity were significantly correlated inversely with age. Thus the reason why we could not detect a significant increase in NK activity among quitters might be explained partly by our elder study subjects than those in the previous study.

Inconsistent results were observed on the relationship between the change in NK activity and exposure levels from smoking assessed with cigarettes consumed per day, smoking index, or urine cotinine in this study. We believe that urine cotinine is a more reliable marker for quantifying the intake of nicotine from smoking than the other measurements because cotinine is a main proximate metabolite of nicotine and, in general, urine cotinine levels are well correlated with plasma cotinine concentration^14^^)^. Study subjects with higher levels of urine cotinine at baseline might have more increased NK activities after smoking cessation.

Our data show that NK activity tended to decrease from the baseline among cigarette smokers. This decrease might be caused by a seasonal variation of NK activity. If this is the case, the changes in NK activity among cigarette smokers may be taken as those of controls: NK activity among quitters was interpreted to show substantial increases compared with that among cigarette smokers. Another plausible interpretation is mental stress with the failure of quitting smoking. Previous reports have showed the association between low NK cell activity and mental distress (i.e., major depression, loneliness, and life event stress)^15^^, ^^16^^)^ or high levels of mental stress (i.e., not keeping mental stress levels moderate)^10^^, ^^11^^)^. It is suggested that smoking cessation interventions should be the provision of enough effective psychological and behavioral therapies for smoking cessation not to discourage cigarette smokers even if they cannot cease smoking.

Our 6 months’ duration of follow-up might be too short to assess the relationship between smoking cessation and NK activity even though the duration in our study was longer than in the previous study. Given the potential implications of this study, the following studies should consider the age and smoking exposure levels of subjects, along with the longer follow-up duration after smoking cessation.
